# Efficacy and safety of ultrasound-guided percutaneous microwave ablation for hepatocellular carcinoma at specific anatomic sites of the liver: a systematic review and meta-analysis

**DOI:** 10.3389/fsurg.2025.1587435

**Published:** 2025-05-22

**Authors:** Jiandong Zha, Fang Zhang, Tuoyu Cao, Shasha Li, Xuelian Shen, Liangliang Xu, Wenqi Chen

**Affiliations:** ^1^ Department of Clinical Oncology, The University of Hong Kong-Shenzhen Hospital, Shenzhen, Guangdong, China; ^2^ Department of Ultrasound Medicine, The Eighth Affiliated Hospital of Sun Yat-sen University, Shenzhen, Guangdong, China; ^3^ Department of Ultrasound, Peking University Shenzhen Hospital, Shenzhen Key Laboratory for Drug Addiction and Medication Safety, Institute of Ultrasound Medicine, Shenzhen-PKU-HKUST Medical Center, Shenzhen, Guangdong, China

**Keywords:** ultrasound guidance, microwave ablation, liver cancer, specific anatomical sites, meta-analysis

## Abstract

**Objective:**

This meta-analysis aims to assess the efficacy and safety of ultrasound-guided percutaneous microwave ablation (MWA) in the treatment of hepatocellular carcinoma (HCC) located at specific anatomic sites of the liver with those of non-specific sites.

**Methods:**

A systematic search was conducted across five databases, covering the period from the establishment of each database to September 30, 2024. Prospective and retrospective studies involving ultrasound-guided percutaneous MWA for the treatment of HCC were included. Data extraction and statistical analysis were performed using Stata 15.1 software. The main outcomes were 1-year, 3-year, and 5-year overall survival rates, complete ablation rates, and major complication rates. The results were presented as odds ratios (OR) with 95% confidence intervals (CI).

**Results:**

A total of 9 studies were included, involving 2,381 patients, of which 1,047 had HCC at specific anatomic sites, and 1,334 had HCC at non-specific sites. The OR values (95% CI) for overall survival at 1 year, 3 years, and 5 years for patients with HCC at specific anatomic sites compared to non-specific sites were 0.89 (0.59, 1.35), 0.83 (0.66, 1.05), and 1.12 (0.91, 1.38), respectively. The OR for complete ablation rate was 0.97 (0.61, 1.53), and the OR for major complications was 1.44 (0.59, 3.51).

**Conclusion:**

Ultrasound-guided percutaneous MWA for HCC at specific anatomic sites shows similar efficacy and safety to that at non-specific sites, with no significant differences in survival rates, complete ablation rates, or complication rates.

## Highlights

• Ultrasound-guided percutaneous MWA is effective for HCC in special anatomical liver locations, comparable to non-special locations.
• No significant differences in 1-year, 3-year, and 5-year survival rates between special and non-special location groups.
• MWA's advantages include reduced heat sink effect, higher temperatures, and applicability in complex anatomical areas.
• Auxiliary techniques and individualized ablation plans enhance efficacy and safety for treating HCC in special locations.
• Findings support expanding MWA use in patients unsuitable for surgery or those refusing surgery.

## Introduction

Hepatocellular carcinoma (HCC) is the fifth most common malignancy worldwide, accounting for approximately 85%–90% of primary liver cancers. Its incidence and mortality rates continue to rise, making it a major global health issue (1, 2). Although liver transplantation is the optimal treatment, its application is limited by donor shortages. Surgical resection is the preferred treatment (3), but many patients cannot undergo surgery due to impaired liver function or unsuitable tumor location (3). Therefore, there is an urgent need to explore alternative treatment strategies.

Percutaneous thermal ablation techniques, including radiofrequency ablation (RFA), microwave ablation (MWA), and laser interstitial heat therapy, have significantly advanced HCC treatment (4). Radiofrequency ablation is the preferred treatment for small HCCs due to its minimal trauma and fewer complications. The American Association for the Study of Liver Diseases recommends that, for tumors smaller than 2.5 cm, radiofrequency ablation offers similar outcomes to surgical resection (4, 5). However, the efficacy of radiofrequency ablation is limited by the tumor's location, especially when the tumor is near blood vessels, the liver hilum, the gastrointestinal tract, the gallbladder, or the diaphragm. In these areas, the heat sink effect can result in incomplete ablation, with local progression rates reaching as high as 30%. The heat sink effect describes the cooling phenomenon that occurs during thermal ablation procedures, such as MWA or RFA, when the target tissue is near a large blood vessel. The flowing blood within the vessel acts as a heat dissipater, drawing thermal energy away from the ablation zone and potentially compromising treatment efficacy (6, 7).

Microwave ablation offers several advantages, including higher temperatures, faster heating rates, and reduced heat sink effects (8). Unlike radiofrequency ablation, microwave conduction is unaffected by tissue drying and carbonization, making it more suitable for complex anatomical sites (8). Recent meta-analyses have shown no significant differences between radiofrequency ablation and microwave ablation in terms of complete ablation rate, 5-year survival rate, and local recurrence rate. However, microwave ablation has shown superior outcomes in reducing distant recurrence and improving 5-year disease-free survival (9). Ultrasound-guided microwave ablation, through real-time monitoring, has become an important approach for treating HCC at specific anatomic sites (9).

Although studies have shown similar rates of local tumor progression in ultrasound-guided microwave ablation for HCC at specific and non-specific sites, there remains a risk of incomplete ablation due to the tumor's proximity to vital organs (10, 11). For example, subphrenic tumors, which are difficult to locate due to respiratory movement, are traditionally considered unsuitable for ablation due to risks such as diaphragmatic perforation and hernia (12, 13).

In light of the above, this study aims to systematically evaluate the efficacy and safety of ultrasound-guided percutaneous microwave ablation for HCC at specific liver sites. Through a comprehensive analysis of existing literature, the goal is to provide strong evidence to support the optimization of non-surgical treatment strategies and the improvement of patient prognosis.

## Methods

### Retrieval strategy

To comprehensively collect literature on ultrasound-guided percutaneous microwave ablation (MWA) for hepatocellular carcinoma (HCC) at specific anatomical sites. Special anatomical locations has been explicitly defined as follows: “tumors within 5 mm of the diaphragm, liver capsule, gallbladder, gastrointestinal tract, hepatic hilum, or within 5 mm of major blood vessels (e.g., branches of the portal vein, hepatic veins, inferior vena cava)”. We conducted systematic searches in multiple databases, including PubMed, Web of Science, Cochrane Library, CNKI, and Wanfang, covering publications up to September 30, 2024. The search end date was intentional to include the most recent preprints and unpublished data available at the time of our search. We used a combination of controlled vocabulary and free-text terms such as “Carcinoma, Hepatocellular”, “Hepatocellular Carcinomas”, “microwave ablation”, “MWA”, and “ultrasou*”. Wildcards were applied to capture variations of the terms. Additionally, references of included studies were manually reviewed to ensure comprehensive coverage.

### Inclusion and exclusion criteria inclusion criteria

#### Inclusion criteria

Eligible studies included patients diagnosed with HCC located in both specific and non-specific liver regions. A specific site was defined as a tumor located within 5 mm of the diaphragm, liver capsule, gallbladder, gastrointestinal tract, hepatic portal, right kidney, or heart, or within 5 mm of a major blood vessel (e.g., portal vein branch, hepatic vein, inferior vena cava). The 5-mm threshold was chosen because tumors in such close proximity to these structures pose higher risks for thermal injury, incomplete ablation, or damage to adjacent organs, and this distance has been referenced in prior studies evaluating technical challenges and complication rates in ablation procedures (14, 15). Studies comparing ultrasound-guided percutaneous MWA for specific (experimental group) and non-specific (control group) sites of HCC were included, ensuring identical interventions in both groups. Primary outcomes included 1-year, 3-year, and 5-year survival rates, complete ablation rate, and safety outcomes such as complications. Eligible study designs were prospective and retrospective cohort studies.

#### Exclusion criteria

Studies were excluded if they involved benign liver tumors, pediatric populations, or MWA combined with other treatments (e.g., systemic chemotherapy, radiofrequency ablation). We also excluded studies with incomplete data, fewer than 20 cases, or lacking a control group. Additionally, reviews, case reports, meta-analyses, conference abstracts, animal studies, and AI-assisted ablation studies were not considered.

### Study selection, data extraction, and quality assessment

The study selection process followed a systematic approach. A literature search was conducted across five databases (PubMed, Web of Science, Cochrane Library, China National Knowledge Infrastructure, and WanFang), followed by the removal of duplicate records. Two independent researchers screened titles and abstracts, excluding irrelevant or ineligible studies. Full-text assessments were then performed to further exclude studies that did not meet inclusion criteria. Any disagreements during the selection process were resolved through discussion or adjudicated by a third reviewer, adhering strictly to Preferred Reporting Items for Systematic Reviews and Meta-Analyses (PRISMA guidelines) (16).

Data extraction was also performed independently by Researcher A and Researcher B. Key information collected from each study comprised the first author's name, publication year, study design, patient characteristics (including sample size, age, gender distribution, and specific inclusion/exclusion criteria), and intervention details (such as the MWA device used, power settings, ablation duration, and any auxiliary techniques like artificial ascites). We also recorded relevant outcomes, including 1-year, 3-year, and 5-year overall survival, complete ablation rate, and incidence of major complications (e.g., hemorrhage, biliary leakage, infection). In cases of missing or ambiguous data, attempts were made to contact the corresponding authors for clarification. Discrepancies in data extraction were resolved by re-evaluating the full text or consulting a third reviewer when necessary.

The risk of bias in each study was assessed using the Newcastle-Ottawa Scale (NOS) (17). This tool evaluates the selection of study groups, the comparability of groups, and the ascertainment of outcomes, assigning a maximum of nine stars. A total score of six or above generally indicated moderate-to-high quality, whereas scores below six suggested a higher risk of bias. Both researchers independently assigned NOS ratings and documented the rationale for their judgments. Any disagreements were addressed through discussion, or if needed, adjudicated by a third reviewer. Studies with potential sources of bias, such as limited follow-up or unaddressed baseline imbalances, were closely scrutinized during sensitivity analyses to ensure the robustness of the meta-analysis findings.

### Statistical analysis

Statistical analysis was performed using Stata 15.1 software (18, 19). For binary outcomes, such as survival rates, complete ablation rate, and complication rates, odds ratios (ORs) and their 95% confidence intervals (CIs) were calculated. Heterogeneity was assessed using the Cochrane Q test and *I*^2^ statistics, where *I*^2^ ≤ 25% indicated low heterogeneity, 25% < *I*^2^ ≤ 50% indicated moderate heterogeneity, and *I*^2^ > 50% indicated high heterogeneity. When *I*^2^ ≤ 50% and *P* ≥ 0.10, the fixed-effect model was used; otherwise, the random-effect model was adopted. The robustness of the results was further assessed through Duval and Tweedie's trim-and-fill analysis (20). Publication bias was evaluated using the Egger linear regression test, with *P* ≥ 0.05 indicating a low risk of bias (21). All statistical tests were two-sided, and the significance level was set at *P* < 0.05. The results were presented in the form of forest plots.

## Results

### Literature search results and characteristics of included studies

**Figure 1:**
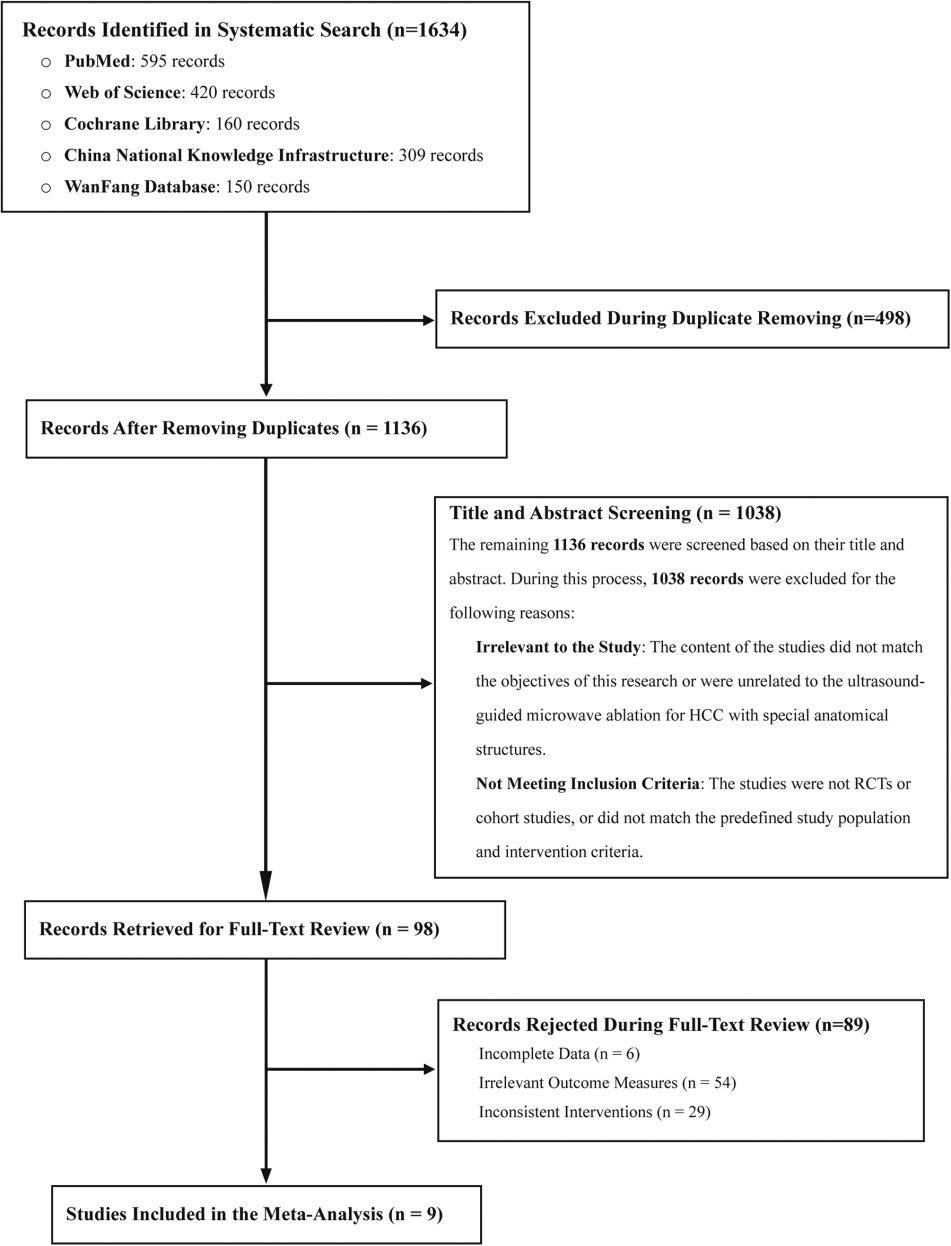
Flow diagram illustrating the literature screening process for identifying eligible studies for inclusion in the meta-analysis.

A total of 1,634 studies were retrieved according to the predefined search strategy. After removing duplicates and excluding studies that did not meet the inclusion criteria, such as studies lacking control groups, reviews, meta-analyses, conference abstracts, and animal studies, 98 studies were included for full-text evaluation. After further assessment, nine studies met all inclusion criteria and were included in the meta-analysis (3, 10, 22–28). The research selection process is shown in Figure 1.

The nine included studies involved a total of 2,381 patients, of whom 1,047 had specific anatomical site HCC and 1,334 had non-specific anatomical site HCC. All studies were cohort studies, some of which were prospective. All patients received ultrasound-guided percutaneous MWA therapy. The primary outcomes were 1-, 3-, and 5-year overall survival rates and complete ablation rates. Secondary outcomes included the incidence of major complications. The characteristics of the included studies are summarized in Table 1 (3, 10, 22–28).

**Table 1:** Baseline characteristics of included studies for meta-analysis.

First author, year	Study desig*n*	Intervention (*n*)	Control (*n*)	Intervention (male/female)	Control (male/female)	Intervention (age, mean ± SD)	Control (age, mean ± SD)	Location of liver cancer in experimental Group	NOS score
Yao et al. (22)	Prospective	142	142	112/30	111/31	55 ± 10	55 ± 13	Subcapsular liver cancer	7
Jin et al. (23)	Retrospective	PSM Before: 23; PSM After: 22	PSM Before: 111; PSM After: 40	PSM Before: 18/5; PSM After: 17/5	PSM Before: 85/26; PSM After: 31/9	PSM Before: 58.9 ± 6.8; PSM After: 58.5 ± 6.6	PSM Before: 57.2 ± 9.3; PSM After: 57.4 ± 10.1	Adjacent to large vessels	9
An et al. (3)	Retrospective	334	155	284/50	132/23	56.8 ± 11.2	58 ± 10.6	Adjacent to large vessels	8
Soliman et al. (24)	Prospective	44	44	34/10	37/7	57 ± 7	59 ± 5.4	Subcapsular, Adjacent to vessels, Adjacent to gallbladder	7
Liu et al. (26)	Prospective	40	40	31/9	NA	55 ± 10	NA	Adjacent to gallbladder, diaphragm, gastrointestinal, large vessels, heart	6
Yang et al. (27)	Retrospective	189	347	139/50	247/66	59.5 ± 10.6	58.4 ± 10.5	Adjacent to large vessels	8
Li et al. (28)	Retrospective	47	82	38/9	65/17	56.7	57.5	Near diaphragm	6
Huang et al. (10)	Retrospective	139	313	NA	NA	NA	NA	Adjacent to large vessels	6
Li et al. (25)	Prospective	89	100	56/33	66/34	60 ± 9.72	58 ± 11.6	Adjacent to diaphragm	7

Abbreviations: *n*, number of participants; Male/Female, gender distribution of participants; Age, Mean ± SD, age of participants expressed as mean ± standard deviation; PSM, propensity score matching; Subcapsular, located beneath the liver capsule; NOS Score, newcastle-ottawa scale score, assessing study quality.

In terms of quality assessment, the Newcastle-Ottawa Scale (NOS) was used. The results showed that all studies were of high quality, with scores of 6 or above. However, some studies had limitations regarding intergroup comparability and follow-up completeness. Detailed quality assessment results are shown in Table 1 (3, 10, 22–28).

## Meta-analysis results

### 1-year overall survival rate

**Figure 2:**
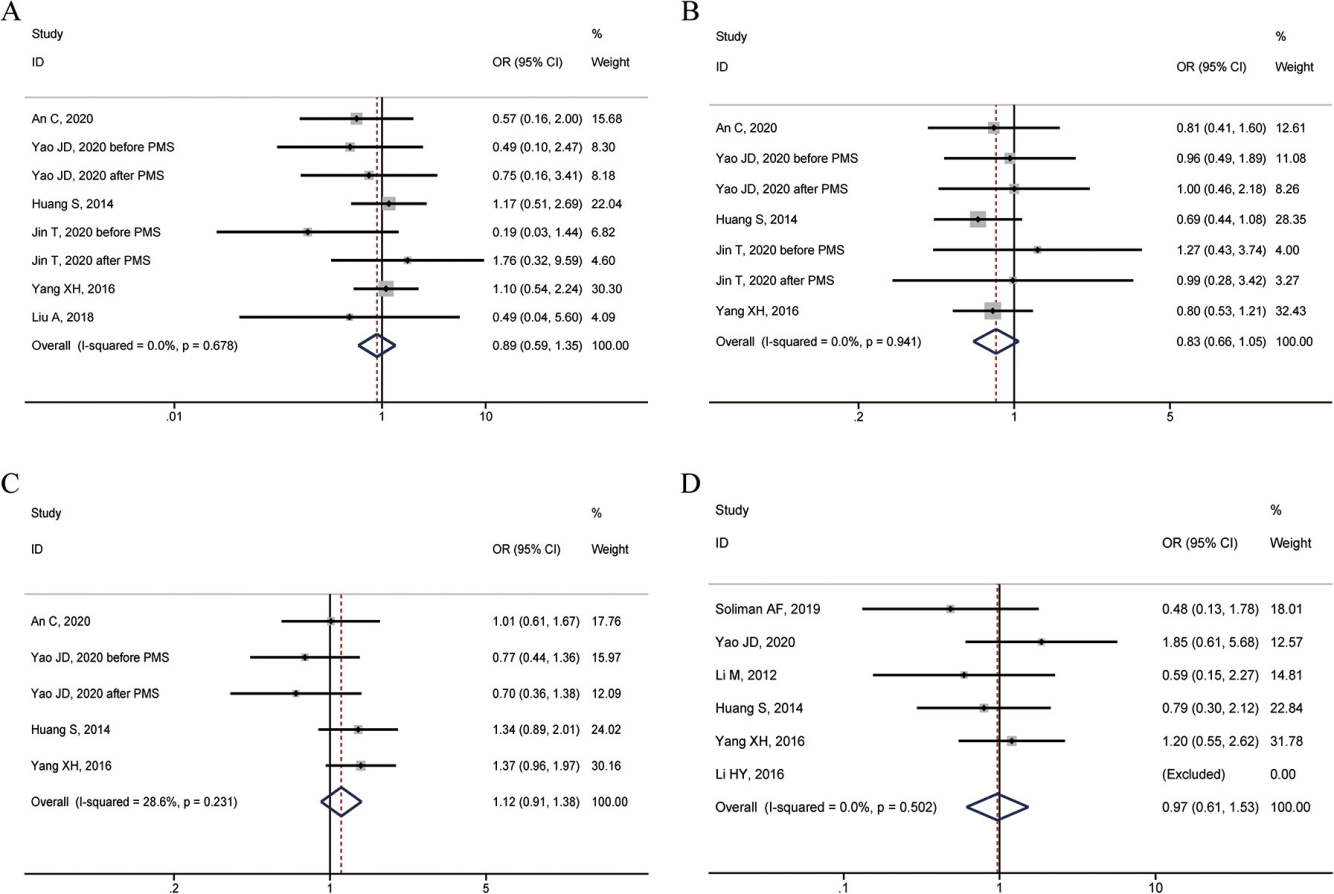
Forest plots of overall survival rates between HCC in special and non-special liver locations. **(A)** 1-year overall survival rate; **(B)** 3-year overall survival rate; **(C)** 5-year overall survival rate; **(D)** Complete ablation rate.

Six studies reported the 1-year overall survival rate. The heterogeneity test showed *I*^2^ = 0.0%, *P* = 0.678, and a fixed-effect model was used. The combined results showed no statistically significant difference in 1-year overall survival between patients with specific and non-specific HCC sites (OR = 0.89, 95% CI: 0.59–1.35) (Figure 2A).

### 3-year overall survival rate

Five studies reported the 3-year overall survival rate. The heterogeneity test showed *I*^2^ = 0.0%, *P* = 0.941, and a fixed-effect model was used. The combined results showed no statistically significant difference in 3-year overall survival between patients with specific and non-specific HCC sites (OR = 0.83, 95% CI: 0.66–1.05) (Figure 2B).

### 5-year overall survival rate

Four studies reported the 5-year overall survival rate. The heterogeneity test showed *I*^2^ = 28.6%, *P* = 0.231, and a fixed-effect model was used. The combined results showed no statistically significant difference in 5-year overall survival between patients with specific and non-specific HCC sites (OR = 1.12, 95% CI: 0.91–1.38) (Figure 2C).

### Complete ablation rate

Six studies reported the complete ablation rate. The heterogeneity test showed *I*^2^ = 0.0%, *P* = 0.502, and a fixed-effect model was used. The combined results showed no statistically significant difference in the rate of complete ablation between HCC patients with specific and non-specific sites (OR = 0.97, 95% CI: 0.61–1.53) (Figure 2D).

### Major complication rate

**Figure 3:**
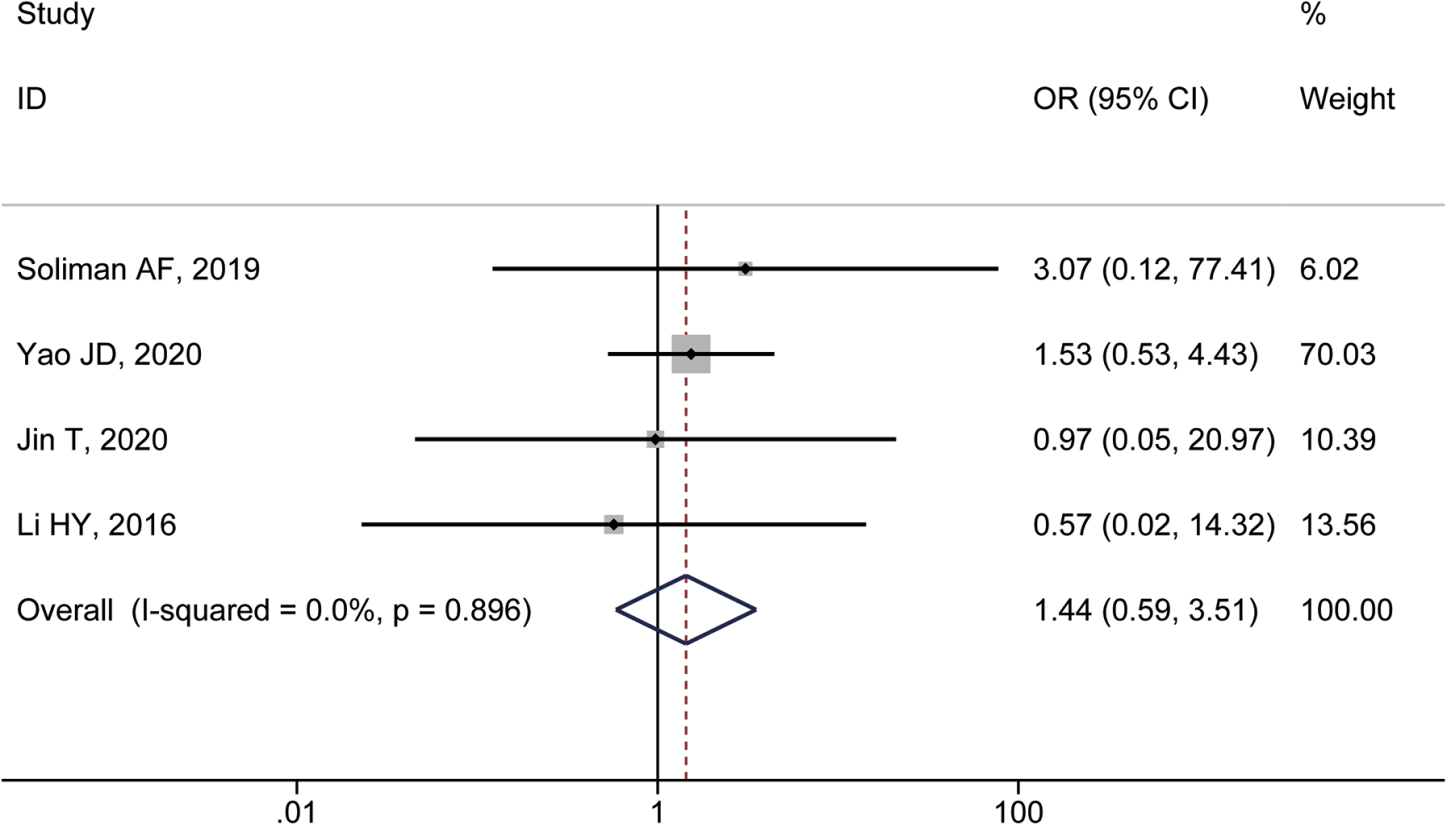
Forest plot comparing the incidence of major complications between HCC patients treated with MWA in special and non-special liver locations.

Four studies reported the incidence of major complications. The heterogeneity test showed *I*^2^ = 0.0%, *P* = 0.896, and a fixed-effect model was used. The combined results showed no statistically significant difference in the incidence of major complications between patients with specific and non-specific HCC sites (OR = 1.44, 95% CI: 0.59–3.51) (Figure 3).

### Publication bias and sensitivity analysis

Publication bias was assessed using the Egger test. The results showed that all *P*-values were greater than 0.05, indicating no significant publication bias (Table 2). Additionally, sensitivity analysis was performed, and the results showed no significant change in the combined effect size, further confirming the robustness of our analysis. This indicates that our conclusions are reliable and stable (Table 2).

**Table 2:** Evaluation of publication bias and sensitivity analysis.

Index	Egger's regression		Duval and tweedie's trim and fill		
	Intercept	*p*	Original effect size	Studies trimmed	Adjusted effect size
1-year overall survival rate	−0.748	0.207	0.89 (0.59, 1.35)	0	0.89 (0.59, 1.35)
3-year overall survival rate	1.043	0.290	0.83 (0.66, 1.05)	3	0.79 (0.57, 1.01)
5-year overall survival rate	−4.914	0.106	1.12 (0.91, 1.38)	0	1.12 (0.91, 1.38)
Complete Ablation Rate	−1.529	0.544	0.97 (0.61, 1.53)	0	0.97 (0.61, 1.53)
Incidence of Major Complications	−0.004	0.966	1.44 (0.59, 3.51)	0	1.44 (0.59, 3.51)

Abbreviation: P, *p*-value (probability value); CI, confidence interval.

## Discussion

This meta-analysis included nine studies on ultrasound-guided percutaneous microwave ablation (MWA) for the treatment of hepatocellular carcinoma (HCC), involving a total of 2,381 patients, of whom 1,047 had HCC at specific anatomical sites and 1,334 had non-specific anatomical site HCC. The results showed no significant differences in 1-year, 3-year, and 5-year overall survival rates, complete ablation rates, and major complication rates between patients with specific and non-specific HCC sites (*P* > 0.05), with low heterogeneity among studies. While the statistical heterogeneity in our analysis was minimal, the possibility of clinical heterogeneity resulting from variations in MWA equipment, power settings, and operator experience, which could influence outcomes. This suggests that the efficacy and safety of ultrasound-guided percutaneous MWA for treating liver HCC at specific anatomic sites is comparable to that at non-specific sites.

These findings contrast with conventional wisdom. Previously, tumors located in specific anatomic areas of the liver, such as those near the diaphragm, gallbladder, gastrointestinal tract, and large blood vessels, were thought to increase the risk of ablation, leading to higher complication rates and reduced outcomes (24). However, the results of this study indicate that survival and complete ablation rates were not significantly affected in patients with specific site HCC who received ultrasound-guided MWA, and the incidence of major complications was not increased. This is consistent with recent studies. For example, Li et al. reported that HCC patients near the diaphragm had local tumor control and survival rates similar to those of patients with non-specific sites after MWA treatment (28). Wang et al. also supported the efficacy of MWA in treating site-specific HCC (29).

When comparing radiofrequency ablation (RFA) and microwave ablation (MWA) for hepatocellular carcinoma, especially in complex or high-risk anatomical locations, MWA presents several theoretical and practical advantages. In particular, MWA is less susceptible to the heat-sink effect caused by blood flow, which can otherwise lead to incomplete ablation in tumors situated near major vessels (30, 31). By generating higher temperatures more quickly, MWA can create larger and more uniform ablation zones, thereby minimizing residual tumor tissue and potentially reducing marginal recurrence (32, 33). Unlike RFA—which relies on electrical conductivity and can be influenced by tissue carbonization and dehydration—MWA's energy delivery is relatively unaffected by these factors, enabling more consistent performance in challenging lesions. These benefits may be especially relevant for tumors in close proximity to critical structures or in regions with substantial vascularity, where rapid and predictable heating is crucial for achieving complete and safe ablation (32, 33). Nonetheless, some studies within this meta-analysis found similar clinical outcomes between RFA and MWA for smaller tumors, indicating that the choice of modality may also depend on tumor size, operator expertise, and equipment availability. Moreover, data remain sparse for large or anatomically complex lesions, underscoring the need for head-to-head comparisons of RFA and MWA in prospective trials with standardized endpoints such as local tumor progression, overall survival, and procedure-related complications. A clearer understanding of each modality's efficacy and safety profile under similar clinical conditions would guide more tailored treatment strategies and improve outcomes for patients with high-risk or anatomically challenging hepatocellular carcinoma.

In this meta-analysis, several studies noted the use of auxiliary or assistive techniques—most commonly, the induction of artificial ascites or pleural effusion—to facilitate safer and more complete ablation of HCC in anatomically challenging regions (34, 35). Among the nine included studies, only four explicitly reported how often such measures were employed, with usage rates ranging from 15% to 40% for tumors near the diaphragm or gastrointestinal tract. This approach can isolate the lesion from surrounding organs, providing thermal insulation and reducing collateral injury, while simultaneously enhancing the clarity of ultrasound imaging for more precise ablation (34, 35). Although there was a general trend suggesting that these techniques lower the risk of complications and improve treatment outcomes, none of the included studies performed a dedicated subgroup analysis to quantify their direct impact on local tumor control or overall survival. Advances in imaging, such as contrast-enhanced ultrasound, CT/MRI fusion, and real-time three-dimensional visualization, have likewise heightened the accuracy of needle placement and real-time monitoring, minimizing adverse events and improving efficacy (34, 36). However, the limited and inconsistent reporting of auxiliary methods in our included studies precluded a robust, quantitative assessment of their precise benefit. Future research that systematically documents the use of artificial ascites, pleural effusion, and other imaging-guided enhancements would help clarify the full potential of these modalities in improving ablative treatment for hepatocellular carcinoma at special anatomical sites.

Improved operating techniques and increased physician experience are also crucial factors. Accurate needle positioning and real-time temperature monitoring ensure adequate tumor ablation while avoiding thermal damage to adjacent normal tissue (37). Individualized ablation protocols, based on tumor size, location, and surrounding anatomy, help select appropriate ablation parameters and needle placement to maximize efficacy (38). These factors collectively contribute to the success of treating specific-site HCC.

The use of combination treatment strategies should not be overlooked. Prior to ablation, transarterial chemoembolization or percutaneous ethanol injection reduces tumor volume and blood flow, enhancing the ablation effect and reducing thermal damage to surrounding tissue (39). This combined approach further improves the therapeutic outcomes for patients with site-specific HCC.

The results of this study have significant implications for clinical practice. First, the study expands the scope of MWA application, showing that ultrasound-guided percutaneous MWA can be safely and effectively used for liver HCC at specific anatomical sites, providing a new treatment option for patients who are not candidates for surgery or who refuse surgery. Second, the study alleviates concerns about the risks of ablation for site-specific HCC and encourages clinicians to adopt MWA with greater confidence, thereby improving patient survival and quality of life. Furthermore, the study demonstrates that the advantages of MWA technology, combined with assistive techniques and individualized protocols, can achieve optimal outcomes in the treatment of site-specific HCC.

Despite the robustness of our findings, this study has several limitations. First, the included studies were primarily prospective and retrospective cohort studies, lacking high-quality randomized controlled trials (RCTs). Such non-randomized designs may introduce selection bias, confounding factors, and variability in outcome assessment, potentially affecting the reliability of the results. Future research would benefit from high-quality RCTs that standardize patient selection, ablation protocols, and follow-up procedures, allowing for more definitive conclusions regarding the efficacy and safety of ultrasound-guided percutaneous MWA for HCC at special anatomical sites. Second, variability across studies in patient characteristics, tumor size, MWA devices, and ablation parameters limited the ability to perform detailed subgroup analyses, which may have impacted the accuracy of the results. Incorporating larger, multicenter cohorts and stratified analyses based on tumor characteristics and underlying liver function could help refine patient selection criteria and optimize therapeutic strategies. Finally, this study focused mainly on survival rates, complete ablation rates, and major complication rates, while neglecting important clinical outcomes such as quality of life, tumor recurrence, and disease-free survival, which are crucial for a comprehensive understanding of patient prognosis. Future studies should incorporate these key clinical endpoints to provide a more holistic evaluation of treatment effectiveness.

## Conclusion

This meta-analysis indicates that the efficacy and safety of ultrasound-guided percutaneous MWA for treating liver HCC at specific anatomical sites is comparable to that for non-specific sites, with no significant differences in survival rates, complete ablation rates, and major complication rates. The advantages of MWA technology, the application of assistive technologies, improved operational skills, and combination treatment strategies all contributed to this outcome. Although this study has some limitations, it provides strong evidence for the effectiveness of MWA in treating site-specific HCC. Future high-quality studies are needed to further validate and refine the use of MWA for treating specific-site HCC, providing a stronger scientific basis for optimizing clinical decision-making and improving patient prognosis.

## Data Availability

The original contributions presented in the study are included in the article/Supplementary Material, further inquiries can be directed to the corresponding author.
